# Erythritol as a Saccharide Multifunctional Electrolyte Additive for Highly Reversible Zinc Anode

**DOI:** 10.3390/nano14070644

**Published:** 2024-04-08

**Authors:** Linjie Li, Zongwei Guo, Shiteng Li, Piting Cao, Weidong Du, Deshi Feng, Wenhui Wei, Fengzhao Xu, Chuangen Ye, Mingzhi Yang, Jing Zhang, Xingshuang Zhang, Yong Li

**Affiliations:** 1 Key Laboratory for High Strength Lightweight Metallic Materials of Shandong Province (HM), Advanced Materials Institute, Qilu University of Technology (Shandong Academy of Sciences), Jinan 250014, Chinayangmingzhi@qlu.edu.cn (M.Y.); jzhang@sdas.org (J.Z.); 2 State Key Laboratory of Biobased Materials and Green Papermaking, Qilu University of Technology (Shandong Academy of Sciences), Jinan 250353, China; 3 Heilongjiang Institute of Technology, College of Materials and Chemical Engineering, Harbin 150006, China; 4 Equipment Department, Sinopec Offshore Oilfield Service Company Shanghai Drilling Division, Shanghai 201208, China

**Keywords:** erythritol, electrolyte additive, zinc anode

## Abstract

Dendrite formation and water-triggered side reactions on the surface of Zn metal anodes severely restrict the commercial viability of aqueous zinc-ion batteries (AZIBs). In this work, we introduce erythritol (Et) as an electrolyte additive to enhance the reversibility of zinc anodes, given its cost-effectiveness, mature technology, and extensive utilization in various domains such as food, medicine, and other industries. By combining multiscale theoretical simulation and experimental characterization, it was demonstrated that Et molecules can partially replace the coordination H_2_O molecules to reshape the Zn^2+^ solvation sheath and destroy the hydrogen bond network of the aqueous electrolyte. More importantly, Et molecules tend to adsorb on the zinc anode surface, simultaneously inhibit water-triggered side reactions by isolating water and promote uniform and dense deposition by accelerating the Zn^2+^ diffusion and regulating the nucleation size of the Zn grain. Thanks to this synergistic mechanism, the Zn anode can achieve a cycle life of more than 3900 h at 1 mA cm^−2^ and an average Coulombic efficiency of 99.77%. Coupling with δ-MnO_2_ cathodes, the full battery delivers a high specific capacity of 228.1 mAh g^−1^ with a capacity retention of 76% over 1000 cycles at 1 A g^−1^.

## 1. Introduction

The development of clean energy and energy storage devices is of great significance for achieving the goal of green and sustainable development in the world [1,2,3]. Among various energy storage devices, lithium-ion batteries (LIBs) have been ubiquitous in every aspect of our society, including electric vehicles and consumer electronics, due to their superior energy density and cycle life [4]. However, the limited availability of lithium resources, high costs, toxic electrolytes, and safety problems hinder its further application to grid-scale energy storage systems. Fortunately, aqueous zinc-ion batteries (AZIBs) can easily circumvent these problems and have been considered a promising candidate due to being low cost, eco-friendly, and very safe [5,6,7]. In particular, the zinc metal anode provides a high theoretical specific capacity (820 mAh g^−1^ or 5824 mAh cm^−3^), low electrochemical potential (−0.76 V vs. SHE), and good water compatibility [8]. Regrettably, the uncontrolled dendrite growth and the water-triggered side reactions on the Zn surface severely affect the reversibility of the Zn anodes, thus hindering the practical application of AZIBs [9].

So far, considerable efforts have been devoted to addressing the dendrite proliferation and the parasitic side reactions on the Zn surface [10]. Common approaches involve coating the Zn anodes with a protective layer, designing 3D current electrode structures to facilitate deposition, employing hydrogel electrolyte or functional separators to regulate Zn^2+^ ion flux, and adding electrolyte additives to change Zn^2+^ solvation structures or modify the electrode/electrolyte interface [11,12]. In comparison, the most viable and cost-effective method is to directly use additives in aqueous electrolytes. For instance, some additives, such as dimethyl sulfoxide [13] and Zn(H_2_PO_4_)_2_ [14] can react with Zn to form a dense, stable interface layer, and inhibit dendrite growth and the side reaction by isolating the contact between active Zn and the bulk electrolyte. Some solvent additives, such as methanol [15], polyacrylamide [16], ethylene glycol [17], triethyl phosphate [18], and 15-crown-5 ether [19] can partially replace solvated water, which is conducive to restraining water-induced side reactions. Additionally, some organic molecules including supramolecular cyclodextrin [20], nettle extract [21], aromatic aldehyde [22], and arginine [23] can be adsorbed onto Zn nuclei to control the crystal plane directional growth. Furthermore, some surfactant additives such as dibenzenesulfonamide [24], perfluorooctanoic acid [25], tetrabutylammonium sulfate [26], and oleic acid [27] can bond to the Zn anode, forming a hydrophobic adsorption layer, which not only blocks the direct contact of H_2_O and active Zn, thus inhibiting the side reactions, but also balances the electric field or ion distribution, thus leading to a uniform Zn deposition. However, a comprehensive evaluation of the physical/chemical properties of additives to adjust the electrolyte and the anode/electrolyte interface environment is still lacking, and the mechanism is still controversial. An efficient additive should simultaneously satisfy three characteristics: (1) It should be more nucleophilic (with a stronger Lewis alkalinity) than H_2_O molecules, which can deplete the water molecules in the carrier solvation sheath, thereby reducing H_2_O penetration and water-triggered side reactions. (2) It should be able to interrupt the hydrogen bond network between water molecules by forming strong hydrogen bonds with H_2_O molecules, thereby reducing the mass transfer rate of H^+^ and OH^−^. (3) It should have a small steric hindrance, which can promote the formation of a solvated sheath and improve the transport rate of charge carriers [28].

In addition, previous studies have rarely considered the price and the preparation methods of the additives because of the small amount used in AZIBs. The expensive price and the complex preparation process reduce the natural price superiority of AZIBs, especially when applied to grid-scale energy storage systems. Recently, an interesting electrolyte additive, saccharide, has attracted extensive attention due to the advantages of low cost, safety, and abundance in nature. For example, Mai’s Group [29] found glucose could simultaneously modulate the solvation structure of the Zn^2+^ and Zn anode-electrolyte interface. Liang’s Group [30] demonstrated that maltose could modulate solvation structures of Zn^2+^ and alleviate side reactions on Zn electrodes. Erythritol (Et) is widely found in nature, such as in fungi, melons, etc., and can also be detected in the eye lens and plasma of animals. It can be prepared by glucose fermentation, is stable at high temperatures, is stable within a wide pH range, and has been widely used in various domains such as food, medicine, and other industries.

Herein, we introduce Et as a multifunctional additive in ZnSO_4_-based electrolytes and systematically reveal its regulatory mechanism for the realization of dendritic-free zinc anode. According to experimental characterization and theoretical simulation calculations, Et molecules can partially replace the coordination H_2_O molecules to reshape the solvation sheath of hydrated Zn^2+^, destroy the hydrogen bond network of the aqueous electrolyte, inhibit hydrogen evolution, and reduce the influence of side reactions. More importantly, Et molecules tend to adsorb on the zinc anode surface, not only inhibiting water-triggered side reactions by isolating water but also promoting uniform and dense deposition by accelerating the Zn^2+^ diffusion and regulating the nucleation size of the Zn grain. Thanks to this synergistic mechanism, the Zn anode can achieve a cycle life of more than 3900 h and achieve an average CE value of 99.77%. The long-cycle stability of the Zn||δ-MnO_2_ battery has been significantly improved, and the capacity retention rate is 76% after 1000 cycles at 1 A g^−1^.

## 2. Results and Discussion

### 2.1. The Structure of Zn^2+^ Solvation Sheath Analysis with the Et Additives

**Figure 1:**
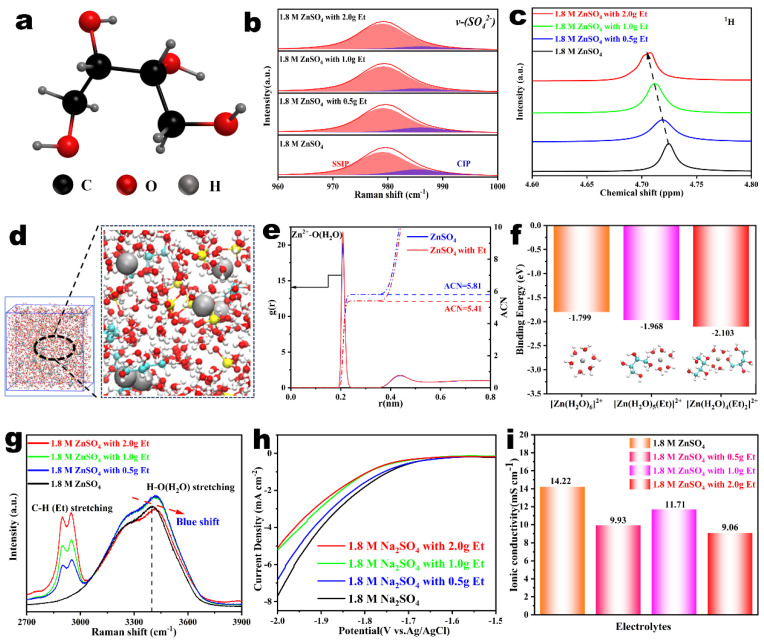
(**a**) Molecular structure of Et. (**b**) Raman spectra (v−SO_4_^2−^ band) and (**c**) ^1^H NMR of 1.8 M ZnSO_4_ electrolytes with various dosages of Et additives. (**d**) Snapshot of the MD simulation of the ZnSO_4_ electrolyte with Et and the local solvation structure of hydrated Zn^2+^. (**e**) Average coordination number between Zn^2+^ and H_2_O in the ZnSO_4_ electrolytes with or without Et. (**f**) Binding energies of original [Zn(H_2_O)_6_]^2+^, [Zn(H_2_O)_5_(Et)]^2+^, and [Zn(H_2_O)_4_(Et)_2_]^2+^ hybrid cluster. (**g**) Raman spectra of 1.8 M ZnSO_4_ electrolytes with various dosages of Et. (**h**) Linear sweep voltammetry plots of the Na_2_SO_4_−based electrolytes with various dosages of Et additives. (**i**) The ion conductivity of 1.8 M ZnSO_4_ electrolytes with various dosages of Et additives.

Figure 1a shows the molecular structure of Et, as a dipole molecule containing multiple hydroxyl groups, Et has stronger zinc affinity compared to H_2_O molecules. It is expected to effectively regulate the structure of the Zn^2+^ solvation sheath and inhibit the activity of H_2_O molecules. Firstly, the variation of the v-SO_4_^2−^ Raman band after the addition of Et additives was investigated to explore the effect of Et molecules on the structure of the Zn^2+^ solvated sheath. According to the association between Zn^2+^ and SO_4_^2−^, there are two forms of solvation structure, solvent-separated ion pair (SSIP, [Zn^2+^(H_2_O)_6_∙SO_4_^2−^]) and contact ion pair (CIP, [Zn^2+^(H_2_O)_5_∙OSO_3_^2−^]). SSIP indicates a weak interaction between Zn^2+^ and SO_4_^2−^, while CIP suggests SO_4_^2−^ has a strong coordination with Zn^2+^ and tends to form an inner-sphere complex [31,32]. As shown in Figure 1b, the SSIP became predominant with increasing additive concentration, while the CIP contribution gradually decreased, which indicates that SO_4_^2−^ hardly affects the structure of the Zn^2+^ solvation sheath due to the strong affinity between Et molecules and Zn^2+^ [33,34]. Nuclear magnetic resonance (^1^H NMR) spectra were further used to confirm the influence of Et on the Zn^2+^ solvation sheath. As shown in Figure 1c, the ^1^H peak of pure H_2_O is located at 4.67 ppm (Figure S1, Supplementary Materials), whereas the introduction of ZnSO_4_ results in a noticeable increase of the chemical shift to 4.73 ppm, showing a lower electronic cloud density of H_2_O due to the tight interaction between Zn^2+^ and H_2_O [35]. The addition of Et will change this trend. As the dosage of Et additives increased to 2.0 g, the ^1^H chemical shift decreased to 4.71 ppm, indicating that the interaction between Zn^2+^ and H_2_O was weakened and the coordinated H_2_O molecules were dehydrated. This phenomenon is attributed to the stronger nucleophilicity of the Et molecules than the water molecules, resulting in strong coordination behavior between Et and Zn^2+^.

Furthermore, a molecular dynamics (MD) simulation and density functional theory (DFT) calculation were carried out to analyze the evolution of the Zn^2+^ solvated sheath structure. Firstly, MD simulation was performed to investigate the Zn^2+^ solvated sheath in different electrolytes. As shown in Figure S2, the Zn^2+^ coordinates with six H_2_O molecules through Zn-O bonds (Zn^2+^-6H_2_O) in the ZnSO_4_ electrolyte. For the Et-containing ZnSO_4_ electrolyte, Et molecules rebuilt the structure of the Zn^2+^ solvate sheath by partially superseding solvated water because of the strong coordination between Zn^2+^ and Et (Figure 1d). Figure S3 shows the radial distribution function (RDF) of Zn^2+^-O(Et) obtained from MD simulation results in the Et-containing ZnSO_4_ electrolyte system. The RDF of the Zn^2+^-O(Et) bond is located at about 0.2 nm, and we calculated that the average coordination number (ACN) value of Zn^2+^ and Et was 0.399, which once again demonstrates our assumptions about the coordination mechanism. The ACN between Zn^2+^ and H_2_O molecules in ZnSO_4_-based electrolytes with/without Et additives was further calculated and shown in Figure 1e. The remarkable peak of the Zn^2+^-O (H_2_O) pair indicates that H_2_O molecules enter into the solvation structure [36,37]. The ACN of Zn^2+^-O(H_2_O) in baseline ZnSO_4_ electrolyte and Et-containing electrolyte were calculated to be 5.81 and 5.41, respectively. The decreased ACN after the addition of Et additives indicates Et molecules can reshape the structure of the Zn^2+^ solvate sheath by partially superseding solvated water. The binding energies of [Zn(H_2_O)_6−m_(Et)_m_]^2+^ hybrid clusters were also calculated by DFT to reveal the strong nucleophilicity of Et molecules. As shown in Figure 1f, the binding energy of [Zn(H_2_O)_6_]^2+^ (−1.799 eV) was lower than that of [Zn(H_2_O)_5_(Et)]^2+^ (−1.968 eV) and [Zn(H_2_O)_4_(Et)_2_]^2+^ (−2.103 eV). A higher binding energy is beneficial for additives to replace H_2_O molecules from the [Zn(H_2_O)_6_]^2+^ clusters [38]. The binding energy of [Zn(H_2_O)_6−m_(Et)_m_]^2+^ hybrid clusters increased as the Et molecules replaced more H_2_O molecules within [Zn(H_2_O)_6_]^2+^, further proving the superior Zn^2+^ affinity of the Et molecules.

Figure 1g shows the Raman spectra of electrolytes with different amounts of Et additive, further indicating the influence of Et. The broad characteristic peaks from 2750 to 3000 cm^−1^ reflect the C-H stretching vibrations of erythritol molecules, while the O-H stretching vibrations of H_2_O and erythritol molecules together facilitate a wide peak from 3000 to 3700 cm^−1^. The blue shift of the O-H peak can be observed with the addition of erythritol molecules, suggesting that the Et additives can effectively disrupt the hydrogen bond network between H_2_O molecules, greatly inhibit the decomposition of water, and reduce related side reactions [39]. The polarizations in various electrolytes were monitored to estimate the inhibiting effect of Et additives on the hydrogen evolution reaction (HER). To exclude the interference of zinc plating, a Na_2_SO_4_-based electrolyte was used instead of the ZnSO_4_-based one during linear sweep voltammetry experiments [40]. Figure 1h shows that the Et-containing electrolytes delivered a higher overpotential than the pure Na_2_SO_4_ electrolyte. Moreover, the increase in overpotential becomes more pronounced as the concentration increases, indicating the excellent suppression effect of Et additives on the HER. Electrochemical impedance spectroscopy (EIS) was performed on various electrolytes (Figure S4), and the corresponding ionic conductivities (*σ*, mS cm^−1^) were calculated from Equation (1)

(1) $$\sigma =\frac{z^{\prime }}{{z^{\prime }}^{2}+{z^{\prime \prime }}^{2}}\cdot \frac{l}{A}$$

where A is the area of the Zn electrode and l is the distance between two electrodes [28,41]. Figure 1i shows that the ion conductivity of the electrolyte slightly decreases after the addition of Et additives. The ion conductivity fluctuates slightly with the increase of Et concentration. We infer that after the addition of Et, some water molecules surrounding the cation in the main solvation sheath are replaced by Et molecules, leading to an increase in free water molecules and a certain improvement in the ion conductivity of the electrolyte. Compared to the improvement in the overall performance of the battery, the loss of ionic conductivity is considered negligible. The histogram of the pH value indicates that the Et additives show almost no effect on the pH value of pure ZnSO_4_ electrolyte (Figure S5). Based on the above test results, it is believed that the comprehensive performance of 2 g additive concentration is the best. Next, the optimal Et additive concentration of 2 g was chosen to explore its role in stabilizing zinc metal anodes.

### 2.2. Effect of Et Additives on Deposition and Diffusion Kinetics of Zn^2+^

**Figure 2:**
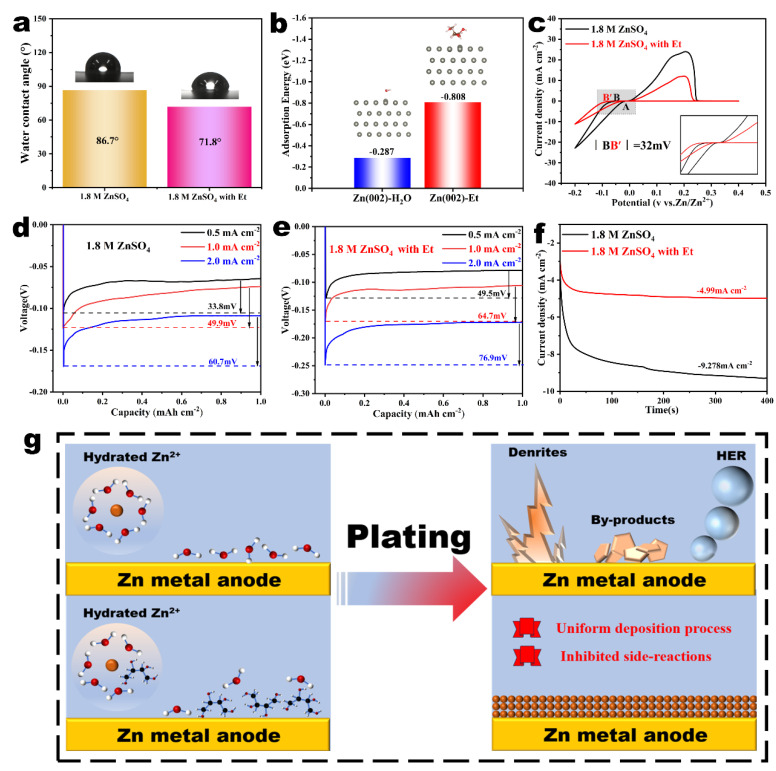
(**a**) Contact angles of ZnSO_4_−based electrolytes with/without Et on bare Zn foils. (**b**) The calculated adsorption energy of H_2_O and Et molecule on Zn(002) surface. (**c**) CV curves of Zn^2+^ nucleation on Cu foil in different electrolytes. Nucleation overpotentials of Zn deposition in (**d**) ZnSO_4_ electrolyte and (**e**) ZnSO_4_ electrolyte with Et at different current densities. (**f**) Chronoamperometry curves of zinc foil in ZnSO_4_ electrolytes with/without Et additives. (**g**) Schematic diagrams of zinc nucleation and growth behavior in ZnSO_4_−based electrolytes with and without Et additives, respectively.

Besides reshaping the structure of the Zn^2+^ solvation sheath, Et molecules also serve a vital role in regulating the interface environment between the zinc mental anode and electrolyte. The wettability of various electrolytes on the Zn electrode was measured by contact angles and the results are presented in Figure 2a. The Et-containing electrolyte had a smaller contact angle with zinc foil (71.8°) than that of the pure ZnSO_4_ electrolyte (86.7°), which suggests that the Et additives are helpful for improving the zinc affinity of the ZnSO_4_ electrolyte. This contributes to the uniform nucleation behavior of Zn electrodes, regulates the distribution of Zn^2+^ on the electrode surface, and has a positive impact on the stability of the electrode. To better understand the adsorption mechanism of Et, the adsorption energy of different molecules on the Zn foil was calculated using density functional theory (DFT) and illustrated in Figure 2b. The adsorption energy of the Et molecule on the Zn(002) plane (−0.808 eV) was significantly larger than that of the H_2_O molecule (−0.287 eV). This suggests that the Et molecules can preferentially adsorb onto the zinc metal electrode and isolate active H_2_O molecules from the Zn anodes, thereby reducing the parasitic side reactions and promoting uniform Zn deposition.

To better understand the mechanism by which Et regulates Zn plating, the cyclic voltammetry (CV) curve of the Zn^2+^ deposition process in different electrolytes was obtained and is shown in Figure 2c. It is evident that the nucleation over-potential after the addition of Et is larger than that of the pristine electrolyte, resulting from the different structure of the Zn^2+^ solvation sheath. The larger radius of the Zn^2+^ solvation sheath and the higher de-solvation energy during electrodeposition increase the electrochemical polarization and ultimately increase the nucleation overpotential [34]. The relationship between the Zn grain radius and the nucleation overpotential of the zinc ion obeys Formula (2)

(2) $$r=2\frac{\gamma V_{m}}{F\eta }$$

where *r* is the radius of the Zn^2+^ nucleation, *γ* is the surface energy of the electrode/electrolyte interface, $V_{m}$ is the molar volume of zinc metal, *F* is Faraday constant (96,485 C mol^−1^), and *η* is the nucleation overpotential [42,43,44]. Therefore, the zinc grain radius is inversely proportional to the nucleation overpotential. The nucleation overpotential in Et-containing electrolytes increased by about 32 mV compared with pure ZnSO_4_ electrolyte, which means that Zn^2+^ in Et-containing electrolytes tends to form in small nuclei, thus promoting the formation of a uniform and dense deposition layer. Furthermore, the nucleation overpotential in ZnSO_4_-based electrolytes with or without Et additives at various current densities were measured and shown in Figure 2d,e. The nucleation overpotential increases with the current density, while the nucleation overpotential of the Et-containing electrolyte is higher than that of the pristine ZnSO_4_ electrolyte under all different current densities, which echoes the above CV analysis. Chronoamperometry measurements were employed to evaluate the Zn nucleation and growth behavior in ZnSO_4_ electrolytes with/without Et additives. For the pristine ZnSO_4_ electrolyte, a long and chaotic in-plane diffusion process of Zn^2+^ cations is exhibited at the initial stage, which is more conducive to the formation of Zn dendrites (Figure 2f) [36]. In contrast, the response current density for the zinc metal anode in the Et-ZnSO_4_ electrolyte delivered a slower downtrend within the first 40 s and then stabilized. This indicates that the diffusion of Zn^2+^ cations switches to 3D diffusion, due to the spatial confinement effect originating from the adsorption of the Et molecules on the zinc surface that shielded the 2D diffusion of the Zn^2+^ cations.

According to the above experimental characterizations and theoretical calculations, we further summarized the mechanism by which Et additives improve the reversibility and stability of the zinc metal electrodes as illustrated in Figure 2g. For pristine ZnSO_4_ electrolyte, Zn^2+^ mainly exists in the form of [Zn(H_2_O)_6_]^2+^, and lots of active H_2_O molecules will accumulate on the zinc metal surface during the Zn plating process, which will accelerate the water-triggered side reactions and eventually lead to an inferior Coulomb efficiency (CE) and affect the reversibility of Zn electrodes. However, the Et molecules can regulate the structure of the Zn^2+^-solvated sheath by replacing partial H_2_O molecules in the solvation sheath. More importantly, the Et molecules can preferentially adsorb on the zinc anode due to the natural zincophilicity, which not only blocks the direct contact of H_2_O and active Zn, thus inhibiting the side reactions, but also balances the electric field or ion distribution, thus leading to a uniform Zn deposition. The above two regulatory mechanisms work together to inhibit the progression of Zn dendrite formation, hydrogen evolution corrosion, and other side reactions, and finally achieve an ultra-stable zinc anode.

### 2.3. The Impact of Et Additives on the Morphology Evolution of Zn Anodes

**Figure 3:**
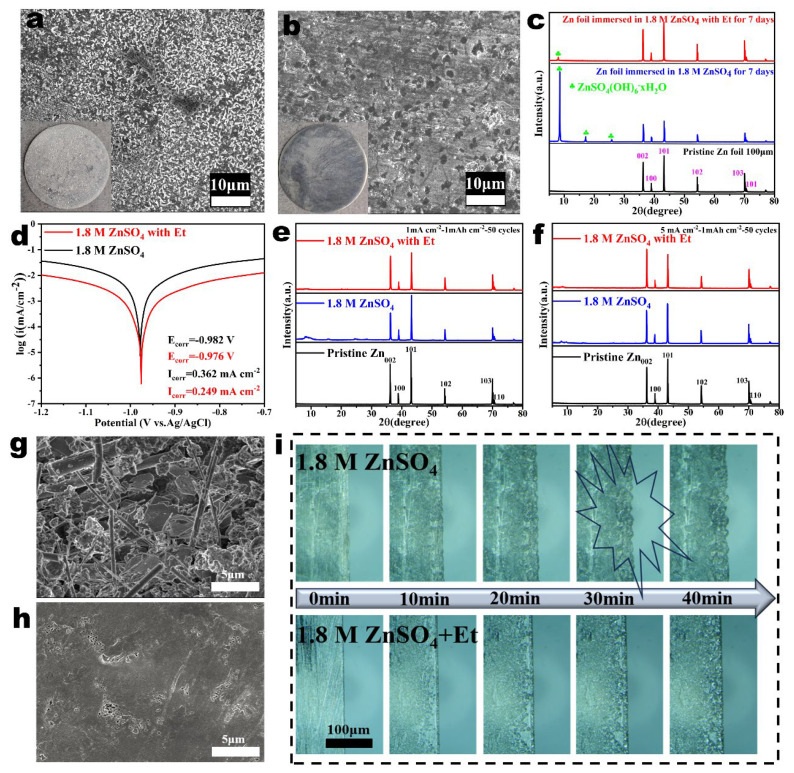
SEM images of the zinc foils after immersion in (**a**) the ZnSO_4_ electrolyte and (**b**) the Et/ZnSO_4_ hybrid electrolyte for a week. (**c**) XRD patterns of zinc foil immersed in ZnSO_4_ electrolytes with/without Et for 7 days. (**d**) Tafel plots of the Zn foil at 1 mV s^−1^ in various electrolytes based on a three-electrode system. XRD patterns of the zinc foils after 50 plating/stripping cycles in different electrolytes at different current densities: (**e**) 1 mA cm^−2^ and (**f**) 5 mA cm^−2^. SEM images of the zinc foils after 50 cycles in (**g**) ZnSO_4_ electrolyte and (**h**) the ZnSO_4_ electrolyte with Et. (**i**) In situ optical observations of Zn deposition morphologies in ZnSO_4_ electrolyte with/without Et additives.

Anti-corrosion performance in aqueous electrolytes is another important parameter used to evaluate the stability of Zn anodes. Therefore, two Zn foils were soaked in ZnSO_4_ electrolyte with or without Et additive for a week, respectively. As shown in Figure 3a, the zinc foil soaked in pristine ZnSO_4_ electrolyte presented a rough surface with numerous cracks and dendrites. For comparison, the zinc foil soaked in the Et/ZnSO_4_ hybrid electrolyte retained a smooth surface as fresh as the pristine one, confirming that the Et additives can obviously promote the corrosion resistance of Zn metal (Figure 3b). As shown in Figure 3c, the XRD patterns can confirm that the by-products appearing on the Zn electrode immersed in ZnSO_4_ electrolyte were mainly composed of zinc hydroxide sulfate species (ZnSO_4_(OH)_6_·xH_2_O). The inhibition effect of Et additives on the corrosion of the Zn electrodes was also evaluated using linear polarization measurements. As exhibited by the Tafel curves (Figure 3d), compared with the Zn foil with pure ZnSO_4_ electrolyte, the Zn foil with Et-containing electrolyte has a higher corrosion potential (−0.982 V to −0.976 V vs. Ag/AgCl) and a lower corrosion current (0.362 mA cm^−2^ to 0.249 mA cm^−2^). In addition, XRD was carried out to analyze the crystal phase on the Zn anodes cycled in different electrolytes (Figure 3e). The intensity ratio between (002) and (101) of Zn electrode with pure ZnSO_4_ electrolyte significantly decreased compared to pristine Zn after 50 cycles at a current density of 1 mA cm^−2^. The enhanced diffraction peak of the (101) plane suggests a longitudinal Zn deposition, which finally results in Zn dendrite growth [45]. In contrast, the Et additives clearly enhance the intensity ratio between (002) and (101), indicating that the Zn metal tends to grow parallel to the substrate, ultimately achieving a dense and uniform deposition layer. Furthermore, the intensity ratio between (002) and (101) was further increased when the current density increased to 5 mA cm^−2^ (Figure 3f).

The surface morphology after Zn deposition in different electrolytes was further compared using SEM. Compared with the pristine zinc foil (Figure S6), the zinc foil cycled in the pristine ZnSO_4_ electrolyte became rough and dispersed numerous flaky dendrites and by-products (Figure 3g). On the contrary, the zinc foil cycled in Et/ZnSO_4_ hybrid electrolyte exhibited an impurity-free surface (Figure 3h). The corresponding cross-sectional SEM images also showed a dense and smooth surface for the Zn electrode cycled in Et-containing electrolyte (Figure S7). To visualize the surface morphology evolution of the zinc anode during plating, a Zn||Zn cell with a quartz inspection window was observed in real-time by in situ optical microscopy. As shown in Figure 3i, some protuberances appeared on the Zn surface of the pristine ZnSO_4_ electrolyte after 20 min. The protuberances continuously grew and evolved into numerous Zn dendrites after 40 min. For the Et-containing electrolyte, no obvious dendrites and bubbles can be observed on the Zn surface during the plating process. The results manifest that the Et additives are beneficial to uniform Zn deposition and the corrosion resistance performance of Zn anodes, thus improving the reversibility of Zn anodes.

### 2.4. Reversibility and Stability Comparison of the Zn Anode in Various Electrolytes

**Figure 4:**
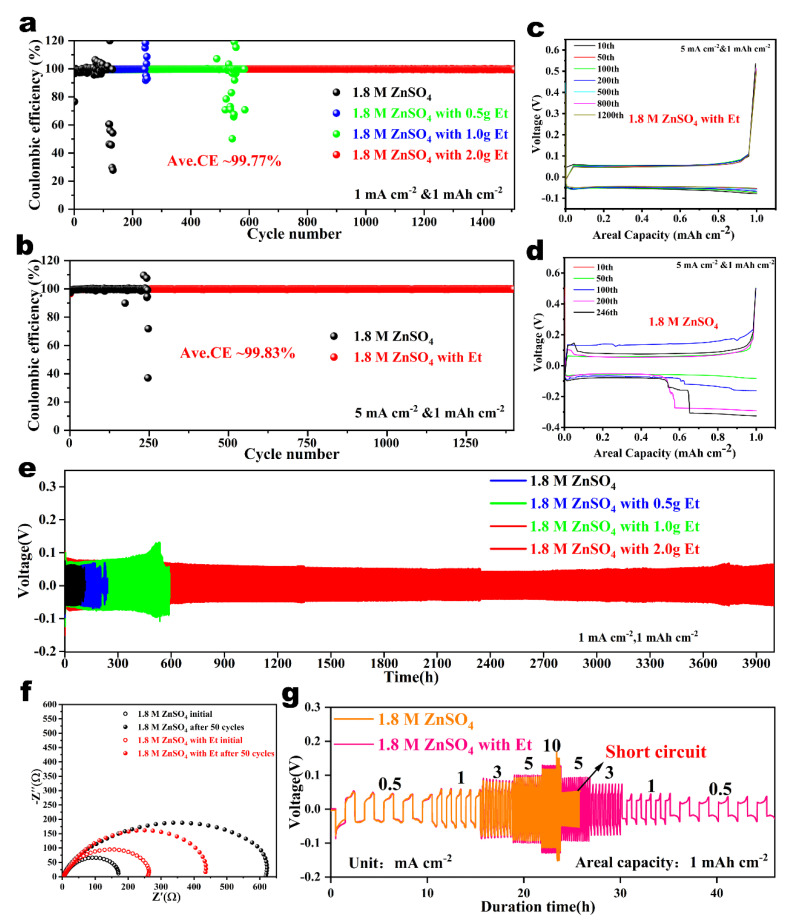
(**a**) CE of Zn plating/stripping at 1 mA cm^−2^ on Cu foils in ZnSO_4_ electrolytes with various dosages of Et additives. (**b**) CE of Zn plating/stripping at 5 mA cm^−2^ on Cu foils in ZnSO4 electrolytes with/without Et additives. Corresponding voltage curve diagrams of (**c**) with Et and (**d**) without Et at 5 mA cm^−2^. (**e**) Cyclic stability of Zn||Zn symmetric cells in ZnSO_4_ electrolytes with various dosages of Et additives at 1 mA cm^−2^. (**f**) The EIS results of Zn||Zn symmetric cells before and after cycling in ZnSO_4_ electrolytes with/without Et additives. (**g**) Comparison of rate capability of Zn||Zn symmetric cells in ZnSO_4_ electrolytes with/without Et additives.

To verify the positive effect of Et additives on the stability of zinc anodes, various half-cells with different electrolytes were prepared. First, the reversibility of Zn plating/stripping was evaluated using a Cu||Zn asymmetric cell. As shown in Figure 4a, the cycle life of the Zn||Cu cell is prolonged with increasing Et content at 1 mA cm^−2^ and 1 mAh cm^−2^. Among them, the cell with the electrolyte containing 2 g Et provides an ultra-long cycle life of over 1500 cycles (3000 h), with an average Coulombic efficiency (CE) of 99.77%. For comparison, the CE of the cell with pristine ZnSO_4_ electrolyte fluctuated violently after 100 plating/stripping cycles, which is caused by local short circuits resulting from severe zinc dendrites. The same trend can be observed when the current density increases to 2 mA cm^−2^ (Figure S8). The cell with an Et-containing electrolyte operated over 1760 plating/stripping cycles (1760 h) with an average CE of 99.81%. In contrast, the cell with pristine ZnSO_4_ electrolyte showed a higher voltage hysteresis and failed after 300 plating/stripping cycles (Figure S9). In addition, even at an ultra-high current density of 5 mA cm^−2^, the cell with Et-containing electrolyte can remain stable over 1300 cycles (520 h), confirming the excellent reversibility of Zn anodes (Figure 4b). However, the CE of the cell with pristine ZnSO_4_ electrolyte showed a sharp fluctuation after 200 cycles, and the polarization increased significantly (Figure 4c,d).

The inhibitory effect of Et additives on dendritic growth can be further demonstrated through the long-term cycling stability of Zn||Zn symmetric cells. As shown in Figure 4e, the cycle life of the symmetric cell is prolonged with the increase of Et content at 1 mA cm^−2^ and 1 mAh cm^−2^; in particular, the cell with 2 g-Et/ZnSO_4_ electrolyte has an ultra-long cycle life of over 3900 h. In contrast, the cell with pristine ZnSO_4_ electrolyte short-circuited at about 120 h. And the same trend can be observed when the current density increases to 5 mA cm^−2^ (Figure S10). Figure 4f shows that the charge transfer impedance (R_ct_) of the cell with Et-containing ZnSO_4_ electrolyte was larger than that of pristine ZnSO_4_ electrolyte at the initial stage due to the adsorption of Et molecules on the Zn surface. Both the R_ct_ of the cells increased as the plating/stripping cycles increased, and the increase in R_ct_ was significantly smaller for the cell with an Et-containing electrolyte compared to that with pure ZnSO_4_ electrolyte. This can be attributed to the fact that the Et-containing electrolyte can form a denser and more uniform Zn deposition layer and can effectively inhibit the generation of by-products. Furthermore, the change in thickness of the cell with an Et-containing electrolyte after cycling was smaller than that in the pristine ZnSO_4_ electrolyte, which confirmed that the Et additives could suppress the HER (Figure S11). Figure 4g shows the rate performance of the symmetric cells in different electrolytes. As the current intensity increases from 0.5 to 10 mA cm^−2^, the Zn||Zn cell with Et electrolyte showed superior cyclic reversibility, while the cell with pure ZnSO_4_ electrolyte short-circuited rapidly. These results demonstrate that Et additives can efficiently stabilize the Zn anode at different current densities, and show superior competitiveness compared with previously reported electrolyte additives (Table S1).

### 2.5. Electrochemical Performance Comparison of AZIBs Equipped with Different Electrolytes

**Figure 5:**
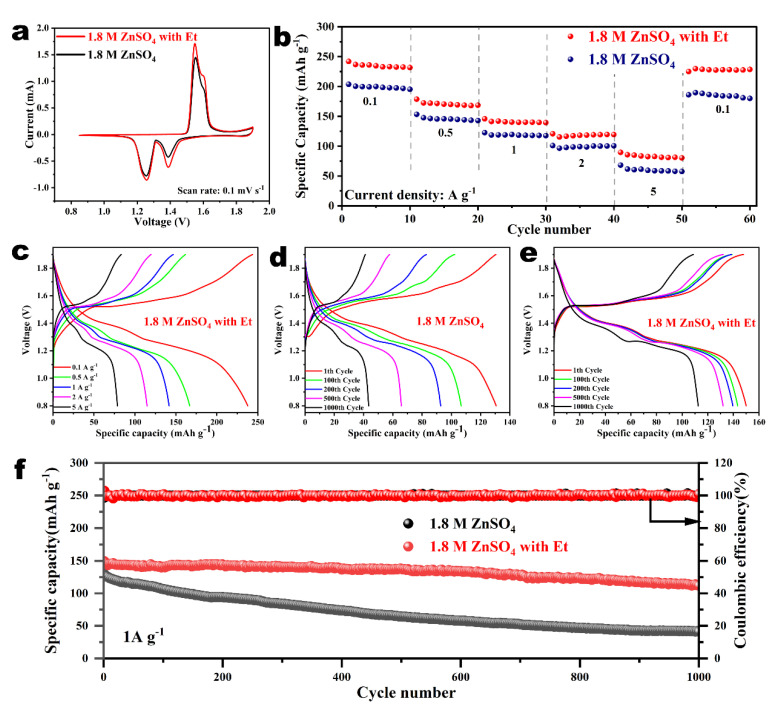
(**a**) CV profiles and (**b**) rate performance of Zn||δ−MnO_2_ full batteries with the electrolytes with/without Et additives. (**c**) Corresponding charge/discharge profiles of full battery in Et−containing electrolyte at different current densities. GCD curves of full batteries with (**d**) pure ZnSO_4_ electrolyte and (**e**) Et−containing electrolyte at 1 A g^−1^. (**f**) Cycling performance of full batteries with different electrolytes at 1 A g^−1^.

The Zn||δ-MnO_2_ full batteries with different electrolytes were assembled to evaluate the practical application of Et additives in AZIBs. The crystal phase and morphology of δ-MnO_2_ cathodes were characterized by XRD and SEM (Figures S12 and S13). As shown in Figure 5a, the full batteries with Et additives showed similar redox peaks to those without Et additives, confirming an analogous electrochemical response. Subsequently, the rate performance of the full batteries was tested within the range of 0.1 to 5 A g^−1^. The Zn||δ-MnO_2_ battery utilizing Et/ZnSO_4_ hybrid electrolyte exhibited a higher capacity compared to that in pristine ZnSO_4_ electrolyte at various current densities (Figure 5b). The full battery with Et-containing electrolyte can still present an average capacity of 89.7 mAh g^−1^ at 5 A g^−1^, and a high capacity of 228.1 mAh g^−1^ was achieved at 0.1 A g^−1^. Figure 5c and Figure S14 show the charge/discharge profiles of the batteries with or without Et additives at different rates, respectively. These results indicate that Et additives have an improved effect on the stability and durability of the entire battery system.

Figure 5d,e show the corresponding GCD curves of the batteries without or with Et additives at various cycles, respectively. The excellent cycling stability of the full batteries utilizing Et/ZnSO_4_ hybrid electrolyte is attributed to the synergy gains of solvation and interface regulation mechanisms, which can suppress dendrite formation and water-triggered side reactions. Afterward, the cycling performance for the full batteries in different electrolytes at 1 A g^−1^ is illustrated in Figure 5f. It is worth noting that the capacity of the full battery using pristine ZnSO_4_ electrolyte rapidly decayed to 41.3 mAh g^−1^ after 1000 cycles. For comparison, the full battery with an Et-containing electrolyte possessed a remarkable capacity (112.7 mA h g^−1^) and a higher capacity retention (76%) after 1000 cycles. These results indicate that Et as an additive in ZnSO_4_ electrolyte can effectively prolong the cycle life of zinc anodes, providing a new approach to improve the performance of aqueous zinc ion batteries.

## 3. Conclusions

To pursue the high reversibility of Zn metal anodes, erythritol was introduced as a multifunctional additive in ZnSO_4_-based electrolytes. Screening a series of electrolytes with Et shows that Et improves the cycling stability and inhibits side reactions by reshaping the environment of the electrolyte and the anode/electrolyte interface. Combined crystallographic/morphology characterizations, electrochemical analysis, DFT calculations, and MD simulation revealed that Et can reshape the Zn^2+^ solvation structure and tends to adsorb on the zinc anode surface, isolate water, and accelerate Zn^2+^ diffusion. The Zn||Zn symmetric cell employing an Et-containing electrolyte achieves an extremely long cycle life of more than 3900 h at 1 mA cm^−2^ and a high CE of 99.77% over 1500 cycles. In addition, the Zn||δ-MnO_2_ full battery demonstrated superior cycle performance and capacity retention, providing a high capacity of 112.7 mAh g^−1^ after 1000 cycles with a retention rate of 76%. These results show that Et additives can effectively improve the reversibility of zinc metal anodes, and the low-cost and mature preparation process of erythritol is expected to promote the application of AZIBs in grid-scale energy storage systems.

## Data Availability

The data presented in this study are available on request from the corresponding author.

## Supplementary Materials

The following supporting information can be downloaded at: https://www.mdpi.com/article/10.3390/nano14070644/s1; Figure S1. 1H NMR spectrum of pure H_2_O solution; Figure S2. Snapshot of the MD simulation of the ZnSO4 electrolyte and the local solvation structure of hydrated Zn^2+^; Figure S3. RDF for Zn^2+^-O(Et) obtained from MD simulation results in the Et/ZnSO_4_ hybrid electrolyte system; Figure S4. EIS of ZnSO4 electrolytes with various amounts of Et and their enlarged view (inset); Figure S5. pH of ZnSO_4_ electrolytes with various amounts of Et; Figure S6. SEM image of pristine Zn foil; Figure S7. Cross-sectional SEM images of Zn foil cycled in ZnSO4 electrolyte without (a) and with (b) Et after 50 cycles; Figure S8. CE of Zn plating/stripping at 2 mA cm^−2^ and 1 mAh cm^−2^ on Cu foils in ZnSO4-based electrolytes with/without Et; Figure S9. The corresponding voltage curves of Zn plating/stripping at 2 mA cm^−2^ and 1 mA h cm^−2^ on Cu foils in ZnSO_4_-based electrolytes with/without Et; Figure S10. Cycling performance of the Zn||Zn cells in the different electrolytes at 5 mA cm^−2^ and 1 mAh cm^−2^; Figure S11. Comparison of Zn||Zn symmetric batteries’ thickness after cycling at 5 mA cm^−2^ and 1 mAh cm^−2^; Figure S12. XRD pattern of the prepared δ-MnO_2_; Figure S13. SEM image of the prepared δ-MnO_2_; Figure S14. Corresponding galvanostatic charge/discharge curves of full cell with pristine 1.8 M ZnSO4 electrolyte at various current densities; Table S1: Comparison of this work with previously reported electrolyte modification strategies for improving the cycling ability of Zn||Zn symmetrical cells. References [19,29,38,46,47,48,49,50,51,52,53,54,55,56,57,58,59,60,61,62,63,64,65,66,67,68,69,70,71] are also cited in the Supplementary Materials.
