# Supplementary material for: Erythritol as a Saccharide Multifunctional Electrolyte Additive for Highly Reversible Zinc Anode
Source: Nanomaterials (Basel). 2024 Apr 8;14(7):644. doi: 10.3390/nano14070644 (PMC11013137; doi:10.3390/nano14070644)
Supplement: Supplementary file 1 [file nanomaterials-14-00644-s001.zip › nanomaterials-2923404-supplementary.pdf]

nanomaterials

Supplementary Materials

Erythritol as a Saccharide Multifunctional Electrolyte Additive for Highly Reversible Zinc Anode

Linjie Li [1,†], Zongwei Guo [2,†], Shiteng Li [3,†], Piting Cao [4], Weidong Du [1], Deshi Feng [1], Wenhui Wei [1], Fengzhao Xu [1], Chuangen Ye [1], Mingzhi Yang [1], Jing Zhang [1], Xingshuang Zhang [1,*] and Yong Li [1,*]

[1] Key Laboratory for High Strength Lightweight Metallic Materials of Shandong Province (HM), Advanced Materials Institute, Qilu University of Technology (Shandong Academy of Sciences), Jinan 250014, China; yangmingzhi@qlu.edu.cn (M.Y.); jzhang@sdas.org (J.Z.)
[2] State Key Laboratory of Biobased Materials and Green Papermaking, Qilu University of Technology (Shandong Academy of Sciences), Jinan 250353, China
[3] Heilongjiang Institute of Technology, College of Materials and Chemical Engineering, Harbin 150006, China
[4] Equipment Department, Sinopec Offshore Oilfield Service Company Shanghai Drilling Division, Shanghai 201208, China
* Correspondence: xszhang@qlu.edu.cn (X.Z.); yongli@sdas.org (Y.L.)
† These authors contributed equally to this work.

Experimental and Calculation Methods

Materials

Zinc sulfate heptahydrate ($ZnSO_4 \bullet 7H_2O$, AR), potassium permanganate ($KMnO_4$, AR), and manganese sulfate ($MnSO_4$, AR) were purchased from the Aladdin company (Shanghai, China). Commercial Zn foil (thickness: 0.1 mm, 99.9 % purity), Cu foil (thickness: 50 µm, 99.9 % purity), and glass microfiber (Whatman, GF/D) were obtained from Shenzhen Kejing Technology (Guangdong, China). Erythritol (Et) was purchased from Shandong Sanyuan Biotechnology Co., Ltd (Shandong, China). Deionized (DI) water was obtained from an ultrapure purification system in our lab.

Electrolyte preparation

First, 1.8 M $ZnSO_4$ aqueous electrolyte was acquired by dissolving zinc sulfate heptahydrate in DI water. Subsequently, various amounts of Et (0.5 g, 1 g, and 2 g) additive were mixed with 5 mL of the prepared $ZnSO_4$ electrolyte. The resulting mixtures were stirred for 3 h to obtain the uniform hybrid electrolyte.

Materials characterizations

The Raman spectra of various electrolytes were collected by a micro-Raman spectroscopy system (532 nm laser). The solvation structure of Zn^{2+} was measured by a nuclear magnetic resonance spectrometer (^1H NMR, Bruker AVANCE NEO 600M). SEM (FESEM, JEOL JSM-7610FPlus, 15 KV) was employed to analyze the morphology of the samples. Crystalline structures of all samples were characterized with X-ray diffraction (XRD, Rigaku D/MAX-2600) with Cu-Kα (λ = 1.540 Å) radiation. The morphology evolution of Zn anodes with/without the Et additive during the Zn deposition process was observed using an in situ optical microscope, where the current density was set at 10 mA cm^{-2}.

Zn||MnO_2 full battery assembly

The δ-MnO_2 was prepared using a hydrothermal method. In detail, 5 mM $KMnO_4$ was dissolved in 75 mL of DI under vigorous stirring; then, 1.75 mL of concentrated hydrochloric acid was added and stirred at room temperature for 45 min. The obtained solution was stirred to get a homogeneous solution and transferred to a 100 mL Teflon autoclave for hydrothermal synthesis. Subsequently, the autoclaves were put in an electric oven for 8 h at 100 °C; then, the products were filtered, washed with DI and ethanol, and dried at 80 °C overnight. The MnO_2 working electrode was prepared by blending active materials, Super P, and polyvinylidene fluoride in a weight ratio of 7:2:1 using N-methyl-2-pyrrolidone as a solvent. The slurry was coated onto the conductive PE film and dried at 80 °C for 8 h under vacuum. The typical loading of active material was controlled as 1.0~2.0 mg cm^{-2}. CR2032 coin-type cells were constructed coupling with the MnO_2 cathode, glass fiber separator, and Zn anode.

Electrochemical characterization

The plating/stripping tests for the asymmetric and symmetric cells were performed on a Neware battery test system. The cyclic voltammetry (CV), chronoamperometry (CA), linear sweep voltammetry (LSV), and electrochemical impedance spectroscopy (EIS) measurements were conducted on a CHI760E electrochemical workstation (Shanghai CHENHUA, China). Linear sweep voltammetry (LSV) was performed in a three-electrode configuration, where the platinum foil was used as the working electrode and counter electrode, and Ag/AgCl as the reference electrode with a scan rate of 5 mV s^{-1}. The corrosion potential and corrosion current density of the Zn anode were obtained by linearly fitting Tafel plots in a three-electrode configuration, where the Zn foil was used as the working electrode, platinum foil as the counter electrode, and Ag/AgCl as the reference electrode with a scan rate of 1.0 mV s^{-1}. Electrochemical impedance spectroscopy (EIS) of the Zn||Zn symmetrical cell was conducted in a frequency range of 0.01 Hz to 100 kHz. Chronoamperometry (CA) curves were conducted at a constant overpotential of −150 mV for 400 s. Cyclic voltammetry (CV) of the Cu||Zn asymmetric cell was conducted in a voltage range of −0.2–0.4 V (vs. Zn^{2+}/Zn) with a scan rate of 1 mV s^{-1}. The assembled full batteries were tested at a series of current densities with a voltage window of 0.8–1.9 V.

Calculation methods

Molecular Dynamics (MD) Simulation: The partial charge of $ZnSO_4$ and Et molecules was calculated using Gaussian 16 code, and the 6-311g(d, p) basis functions were applied [1]. The OPLSS-AA force field [2] and MKTOP [3] were used to parametrize all atoms, such as the bond parameters, angle parameters, dihedral angles, and so on. In system 1, 90 $ZnSO_4$ molecules and 2778 H_2O molecules were randomly inserted into a 6.0 nm*6.0 nm*6.0 nm cube. In system 2, 90 $ZnSO_4$ molecules, 80 Et molecules, and 2778 H_2O molecules were randomly inserted into a 6.0 nm*6.0 nm*6.0 nm cube. The MD simulations were performed using the GROMACS 2021 software package [4-6]. The steepest descent method was applied to minimize the initial energy for each system with a force tolerance of 1 kJ/(mol^{-1} nm^{-1}) and a maximum step size of 0.002 ps before MD calculations [7]. In all three directions, periodic boundary conditions were imposed. The leapfrog algorithm was used to integrate the Newtonian equation of motion. The MD simulation was processed in an NPT ensemble, and the simulation time was 20 ns. In the NPT simulations, the pressure was maintained at 1 bar by the Berendsen barostat in an isotropic manner [8], and the temperature was maintained by the V-rescale thermostat at 298.15 K. The Particle Mesh Ewald (PME) method with a fourth-order interpolation was used to evaluate the electrostatic interactions, and a cutoff of 1.0 Å was employed to calculate the short-range van der Waals interactions [9].

DFT Calculation: The first-principles tool Vienna Ab initio Simulation Package (VASP6.3.2) [10, 11] was employed to perform all density functional theory (DFT) calculations within the generalized gradient approximation (GGA) using the Perdew–Burke–Ernzerhof (PBE) [12] formulation. We have chosen the projected augmented wave (PAW) potentials [13, 14] to describe the ionic cores and take valence electrons into account using a plane wave basis set with a kinetic energy cutoff of 450 eV. Partial occupancies of the Kohn–Sham orbitals were allowed using the Gaussian smearing method and a width of 0.05 eV. For the optimization of both geometry and lattice size, the Brillouin zone integration was performed with a 0.04 $Å^{-1}$ k-mesh Gamma centered sampling [15]. The self-consistent calculations applied a convergence energy threshold of 10^{-5} eV. The equilibrium geometries and lattice constants were optimized with maximum stress on each atom within 0.02 eV $Å^{-1}$. The 17 Å vacuum layer was normally added to the surface to eliminate the artificial interactions between periodic images. The weak interaction was described with the DFT+D3 method using empirical correction in Grimme's scheme [16, 17]. The adsorption energy was calculated as follows: Eads = E(*adsorbent) - E(*) - E(adsorbent). E(*adsorbent), E(*) and E(adsorbent) represent the total energy of * adsorbent, * and adsorbent molecule, respectively.

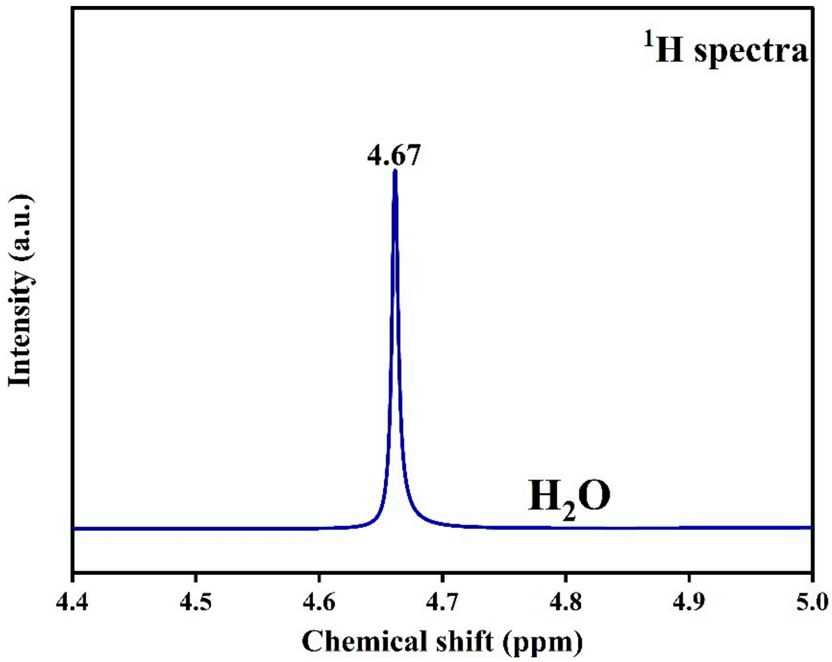

Figure S1. ^{1}H NMR spectrum of pure H_2O solution.

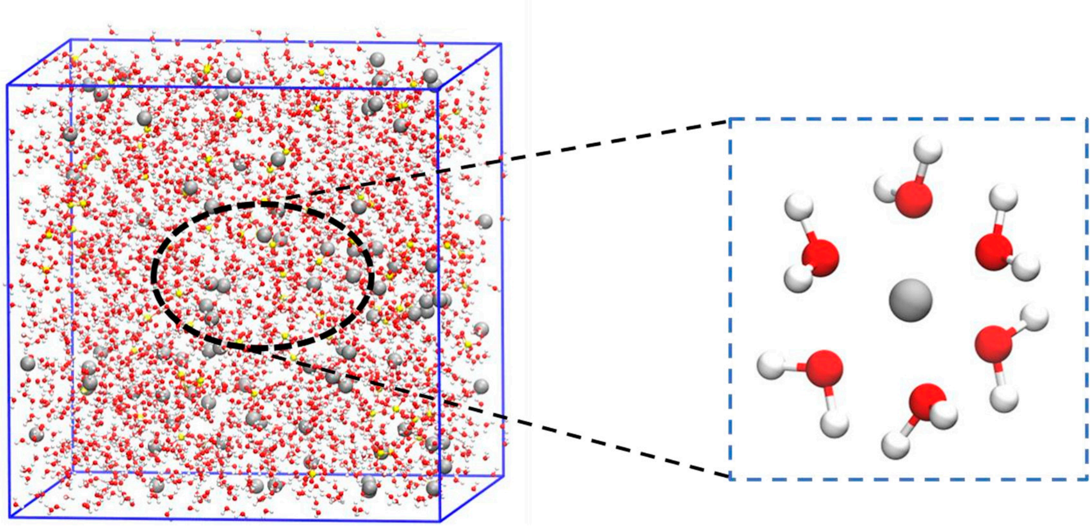

Figure S2. Snapshot of the MD simulation of the $ZnSO_4$ electrolyte and the local solvation structure of hydrated Zn^{2+}.

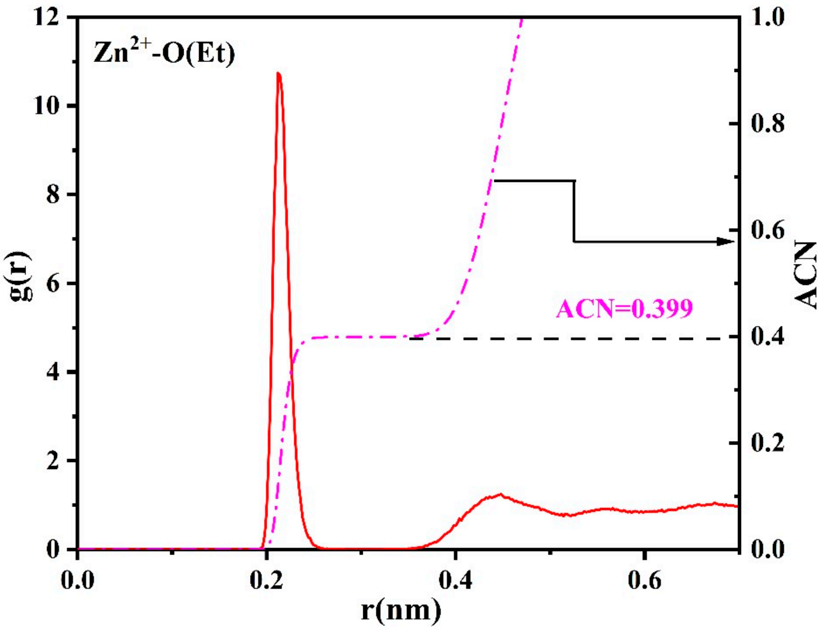

Figure S3. RDF for Zn^{2+}-O(Et) obtained from MD simulation results in the Et/$ZnSO_4$ hybrid electrolyte system.

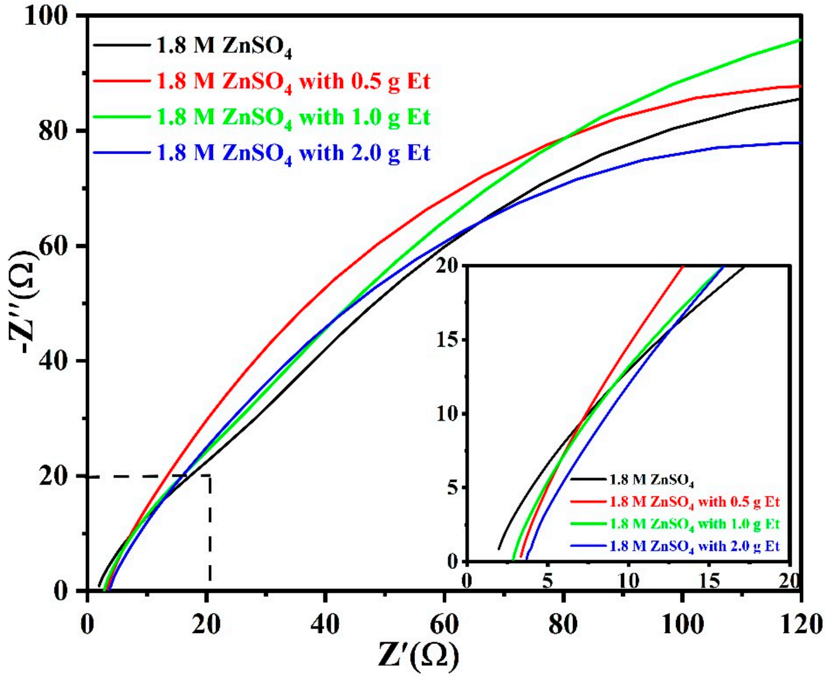

Figure S4. EIS of $ZnSO_4$ electrolytes with various amounts of Et and their enlarged view (inset).

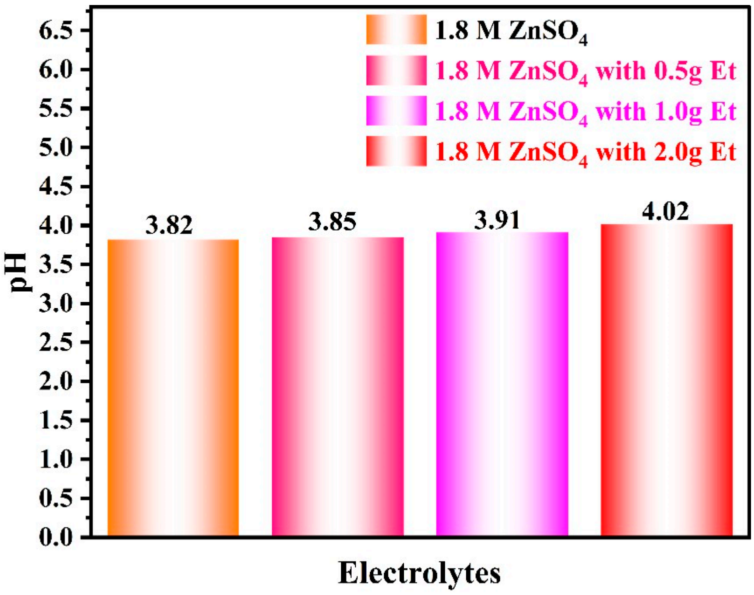

Figure S5. pH of $ZnSO_4$ electrolytes with various amounts of Et.

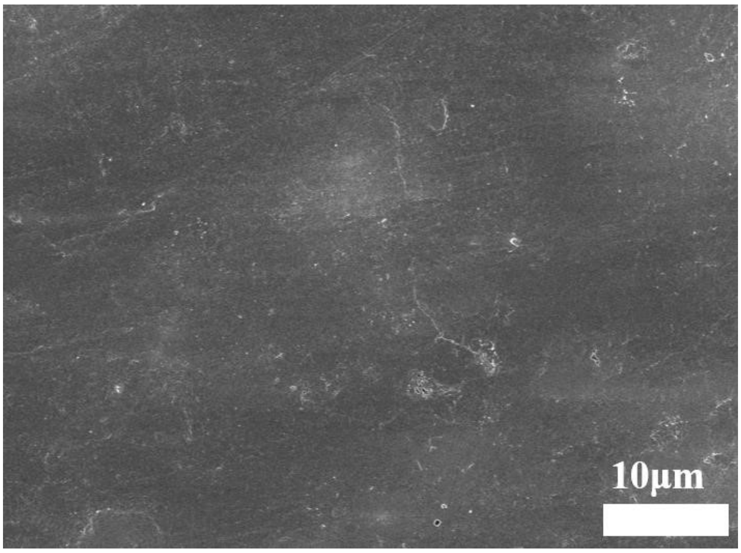

Figure S6. SEM image of pristine Zn foil.

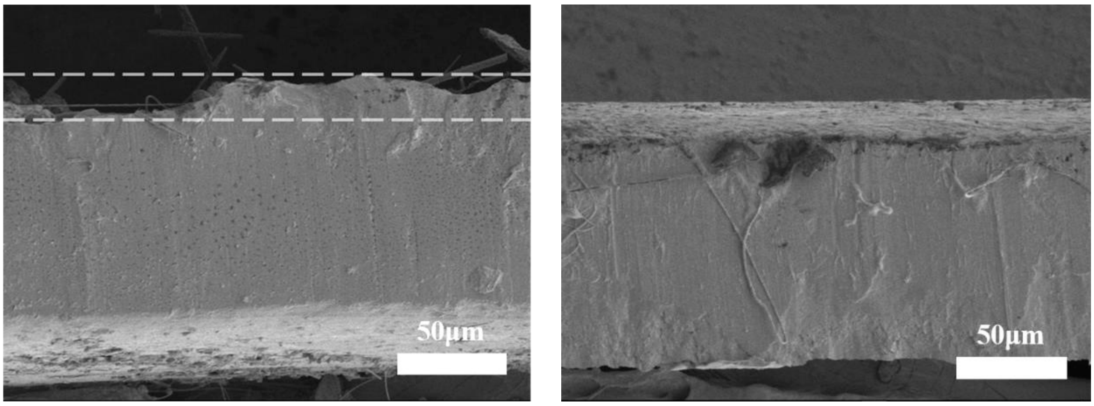

(a) **(b)**

Figure S7. Cross-sectional SEM images of Zn foil cycled in $ZnSO_4$ electrolyte without (**a**) and with (**b**) Et after 50 cycles.

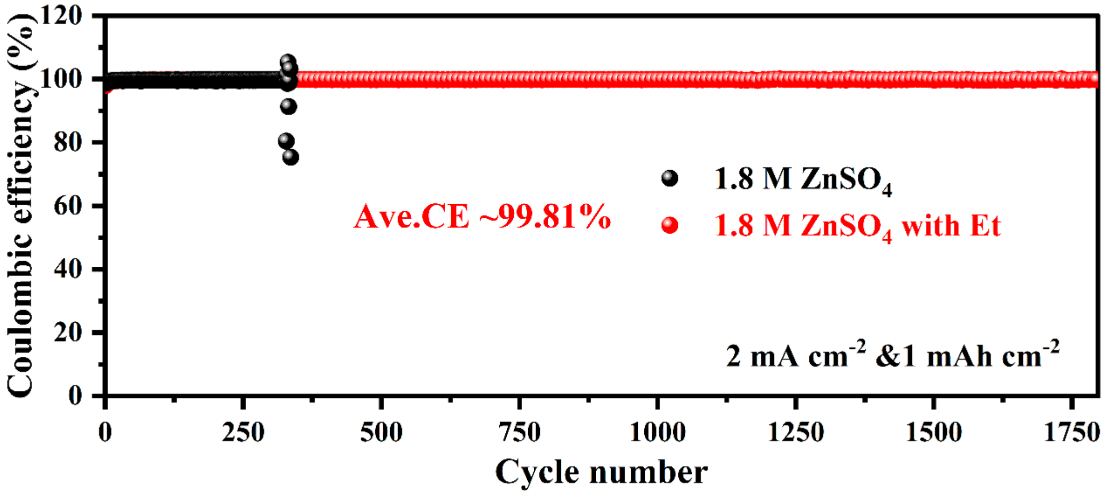

Figure S8. CE of Zn plating/stripping at 2 mA cm^{-2} and 1 mAh cm^{-2} on Cu foils in $ZnSO_4$-based electrolytes with/without Et.

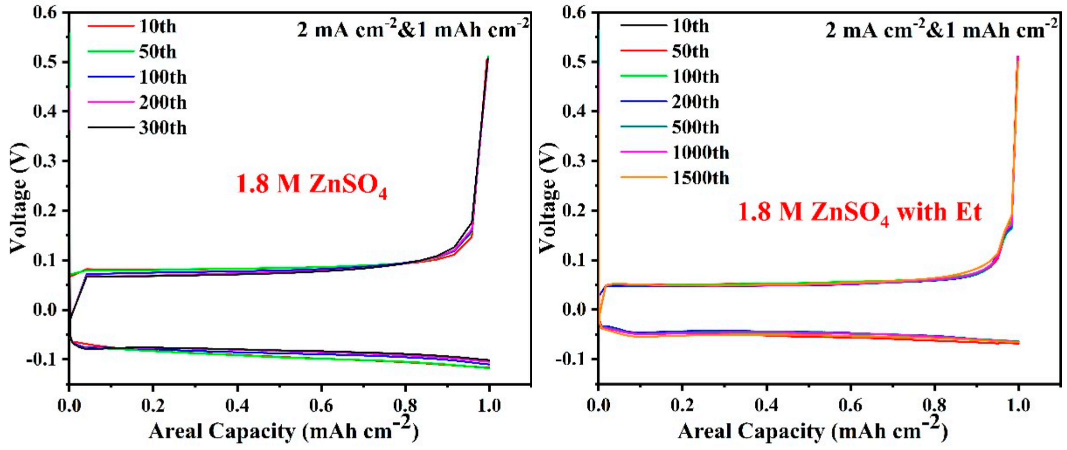

Figure S9. The corresponding voltage curves of Zn plating/stripping at 2 mA cm^{-2} and 1 mA h cm^{-2} on Cu foils in $ZnSO_4$-based electrolytes with/without Et.

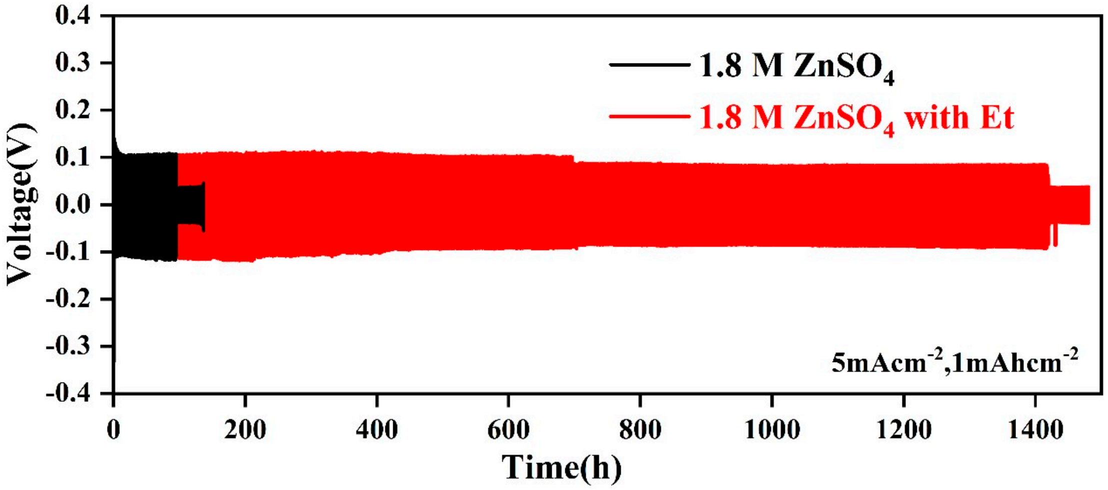

Figure S10. Cycling performance of the Zn||Zn cells in the different electrolytes at 5 mA cm^{-2} and 1 mAh cm^{-2}.

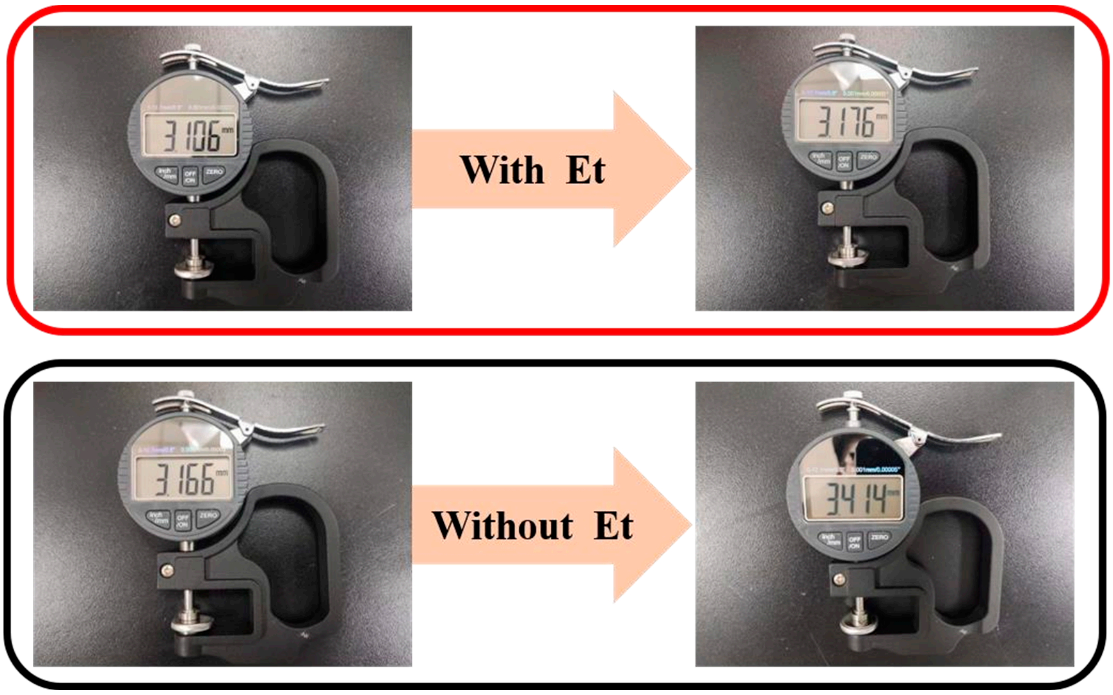

Figure S11. Comparison of Zn||Zn symmetric batteries' thickness after cycling at 5 mA cm^{-2} and 1 mAh cm^{-2}.

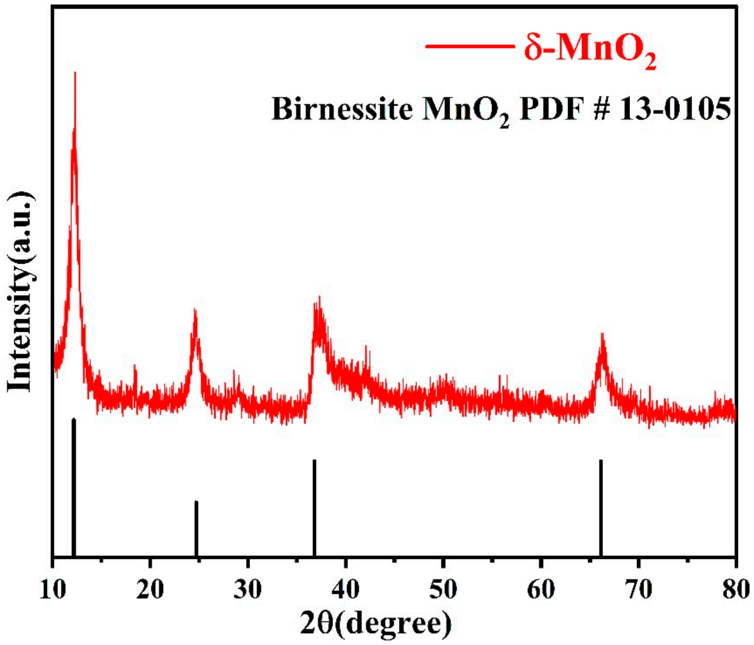

Figure S12. XRD pattern of the prepared δ-MnO_2.

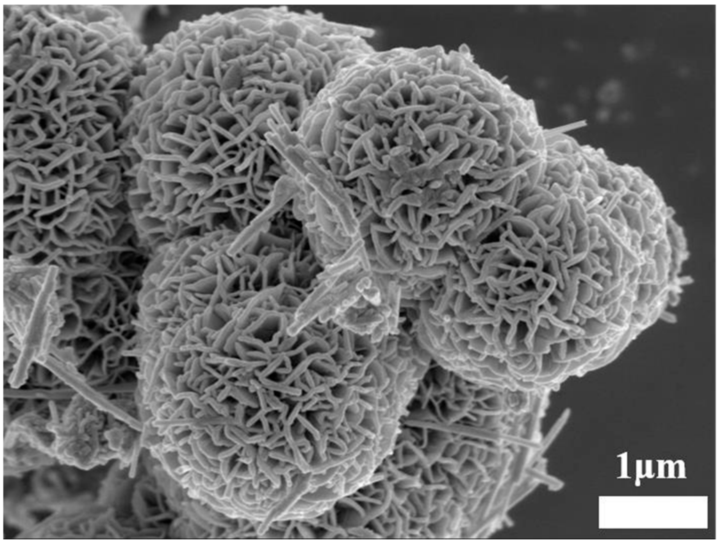

Figure S13. SEM image of the prepared δ-MnO_2.

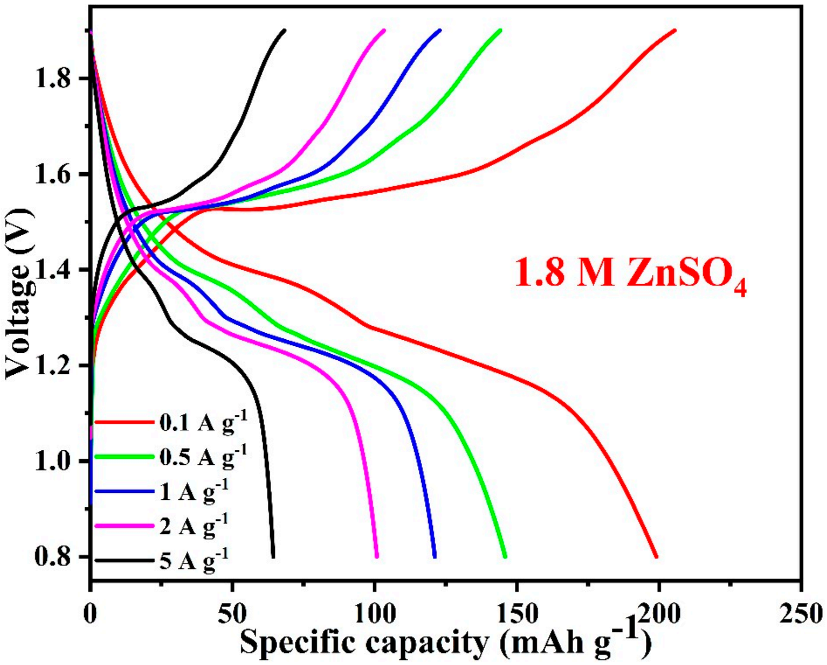

Figure S14. Corresponding galvanostatic charge/discharge curves of full cell with pristine 1.8 M $ZnSO_4$ electrolyte at various current densities.

Table S1. Comparison of this work with previously reported electrolyte modification strategies for improving the cycling ability of Zn||Zn symmetrical cells.

Additive types	Current density/Areal capacity (mA cm^{-2}/mAh cm^{-2})	Cycling life (h)	Reference
Ethylene glycol (EG)	0.5/0.5	1400	[18]
Acetonitrile (AN)	2/2	650	[19]
Arginine (Arg)	0.5/0.5	515	[20]
Glucose	1/1	2000	[21]
Tridentate citrate	5/1.25	400	[22]
Vanillin	1/1	1000	[23]
Glycerol	1/1	1500	[24]
Glycine	1/0.5	2000	[25]
Inositol (Ino)	1/1	1700	[26]
TMA_2SO_4	0.5/0.5	1800	[27]
15-Crown-5	2/2	770	[28]
Tripropylene glycol (TG)	0.5/0.25	2000	[29]
**Et**	**1/1**	**3990**	**This work**
	**5/1**	**1400**	

References

[1] Petersson, G.A.; Al-Laham, M.A. A complete basis set model chemistry. II. Open-shell systems and the total energies of the first-row atoms. *J. Chem. Phys.* **1991**, *94*, 6081–6090. https://doi.org/10.1063/1.2335438.

[2] Jorgensen, W.L.; Maxwell, D.S.; Tirado-Rives, J. Development and Testing of the OPLS All-Atom Force Field on Conformational Energetics and Properties of Organic Liquids. *J. Am. Chem. Soc.* **1996**, *118*, 11225–11236. https://doi.org/10.1021/ja9621760.

[3] Ribeiro, A.A.S.T.; Horta, B.A.C.; Alencastro, R.B.D. MKTOP: A program for automatic construction of molecular topologies. *J. Braz. Chem. Soc.* **2008**, *19*, 1433–1435. https://doi.org/10.1590/s0103-50532008000700031.

[4] Van Der Spoel, D.; Lindahl, E.; Hess, B.; Groenhof, G.; Mark, A.E.; Berendsen, H.J. Gromacs: Fast, flexible, and free. *J. Comput. Chem.* **2005**, *26*, 1701–1718. https://doi.org/10.1002/jcc.20291.

[5] Abraham, M.J.; Murtola, T.; Schulz, R.; Páll, S.; Smith, J.C.; Hess, B.; Lindahl, E. Gromacs: High performance molecular simulations through multi-level parallelism from laptops to supercomputers. *SoftwareX* **2015**, *1*, 19–25. https://doi.org/10.1016/j.softx.2015.06.001.

[6] Berendsen, H.J.C.; van der Spoel, D.; Drunen, R.V. GROMACS: A message-passing parallel molecular dynamics implementation Comput. *Phys. Commun.* **1995**, *91*, 43–56. https://doi.org/10.1016/0010-4655(95)00042-E.

[7] Van Gunsteren, W.F.; Berendsen, H.J.C. A Leap-frog Algorithm for Stochastic Dynamics. *Mol. Simul.* **1988**, *1*, 173–185. https://doi.org/10.1080/08927028808080941.

[8] Hess, B.; Bekker, H.; Berendsen, H.J.C.; Fraaije, J.G.E.M. LINCS: A linear constraint solver for molecular simulations. *J. Comput. Chem.* **1997**, *18*, 1463–1472. https://doi.org/10.1002/(sici)1096-987x(199709)18:12<1463::Aid-jcc4>3.0.Co;2-h.

[9] Darden, T.; York, D.; Pedersen, L. Particle mesh Ewald: An N·log(N) method for Ewald sums in large systems. *J. Chem. Phys.* **1993**, *98*, 10089–10092. https://doi.org/10.1063/1.464397.

[10] Kresse, G.; Furthmüller, J. Efficiency of ab-initio total energy calculations for metals and semiconductors using a plane-wave basis set. *Comput. Mater. Sci.* **1996**, *6*, 15–50. https://doi.org/https://doi.org/10.1016/0927-0256(96)00008-0.

[11] Kresse, G.; Furthmüller, J. Efficient Iterative Schemes for Ab Initio Total-Energy Calculations Using a Plane-Wave Basis Set. *Phys. Rev. B* **1996**, *54*, 11169–11186. https://doi.org/10.1103/PhysRevB.54.11169.

[12] Perdew, J.P.; Burke, K.; Ernzerhof, M. Generalized Gradient Approximation Made Simple. *Phys. Rev. Lett.* **1996**, *77*, 3865–3868. https://doi.org/10.1103/PHYSREVLETT.77.3865.

[13] Kresse, G.; Joubert, D. From ultrasoft pseudopotentials to the projector augmented-wave method. *Phys. Rev. B* **1999**, *59*, 1758–1775. https://doi.org/10.1103/PhysRevB.59.1758.

[14] Blöchl, P.E. Projector augmented-wave method. *Phys. Rev. B* **1994**, *50*, 17953–17979. https://doi.org/10.1103/PhysRevB.50.17953.

[15] Monkhorst, H.J.; Pack, J.D. Special points for Brillouin-zone integrations. *Phys. Rev. B* **1976**, *13*, 5188–5192. https://doi.org/10.1103/PhysRevB.13.5188.

[16] Grimme, S.; Antony, J.; Ehrlich, S.; Krieg, H. A consistent and accurate ab initio parametrization of density functional dispersion correction (DFT-D) for the 94 elements H-Pu. *J. Chem. Phys.* **2010**, *132*, 154104. https://doi.org/10.1063/1.3382344.

[17] Grimme, S.; Ehrlich, S.; Goerigk, L. Effect of the damping function in dispersion corrected density functional theory. *J. Comput. Chem.* **2011**, *32*, 1456–1465. https://doi.org/10.1002/jcc.21759.

[18] Qin, R.; Wang, Y.; Zhang, M.; Wang, Y.; Ding, S.; Song, A.; Yi, H.; Yang, L.; Song, Y.; Cui, Y.; et al. Tuning Zn^{2+} coordination environment to suppress dendrite formation for high-performance Zn-ion batteries. *Nano Energy* **2021**, *80*, 105478. https://doi.org/10.1016/j.nanoen.2020.105478.

[19] Hou, Z.; Tan, H.; Gao, Y.; Li, M.; Lu, Z.; Zhang, B. Tailoring desolvation kinetics enables stable zinc metal anodes. *J. Mater. Chem. A* **2020**, *8*, 19367–19374. https://doi.org/10.1039/d0ta06622b.

[20] Chen, Z.; Chen, H.; Che, Y.; Cheng, L.; Zhang, H.; Chen, J.; Xie, F.; Wang, N.; Jin, Y.; Meng, H. Arginine Cations Inhibiting Charge Accumulation of Dendrites and Boosting Zn Metal Reversibility in Aqueous Rechargeable Batteries. *ACS Sustain. Chem. Eng.* **2021**, *9*, 6855–6863. https://doi.org/10.1021/acssuschemeng.1c01609.

[21] Sun, P.; Ma, L.; Zhou, W.; Qiu, M.; Wang, Z.; Chao, D.; Mai, W. Simultaneous Regulation on Solvation Shell and Electrode Interface for Dendrite-Free Zn Ion Batteries Achieved by a Low-Cost Glucose Additive. *Angew. Chem. Int. Ed.* **2021**, *60*, 18247–18255. https://doi.org/10.1002/anie.202105756.

[22] Wang, N.; Zhai, S.; Ma, Y.; Tan, X.; Jiang, K.; Zhong, W.; Zhang, W.; Chen, N.; Chen, W.; Li, S.; et al. Tridentate citrate chelation towards stable fiber zinc-polypyrrole battery with hybrid mechanism. *Energy Storage Mater.* **2021**, *43*, 585–594. https://doi.org/10.1016/j.ensm.2021.10.004.

[23] Zhao, K.; Liu, F.; Fan, G.; Liu, J.; Yu, M.; Yan, Z.; Zhang, N.; Cheng, F. Stabilizing Zinc Electrodes with a Vanillin Additive in Mild Aqueous Electrolytes. *ACS Appl. Mater. Interfaces* **2021**, *13*, 47650–47658. https://doi.org/10.1021/acsami.1c14407.

[24] Zhang, Y.; Zhu, M.; Wu, K.; Yu, F.; Wang, G.; Xu, G.; Wu, M.; Liu, H.; Dou, S.; Wu, C. An in-depth insight of a highly reversible and dendrite-free Zn metal anode in an hybrid electrolyte. *J. Mater. Chem. A* **2021**, *9*, 4253–4261. https://doi.org/10.1039/d0ta11668h.

[25] Gou, Q.; Luo, H.; Zhang, Q.; Deng, J.; Zhao, R.; Odunmbaku, O.; Wang, L.; Li, L.; Zheng, Y.; Li, J.; et al. Electrolyte Regulation of Bio-Inspired Zincophilic Additive toward High-Performance Dendrite-Free Aqueous Zinc-Ion Batteries. *Small* **2023**, *19*, 2207502. https://doi.org/10.1002/smll.202207502.

[26] Ji, H.; Han, Z.; Lin, Y.; Yu, B.; Wu, D.; Zhao, L.; Wang, M.; Chen, J.; Ma, Z.; Guo, B.; et al. Stabilizing zinc anode for high-performance aqueous zinc ion batteries via employing a novel inositol additive. *J. Alloys Compd.* **2022**, *914*, 165231. https://doi.org/10.1016/j.jallcom.2022.165231.

[27] Cao, H.; Huang, X.; Liu, Y.; Hu, Q.; Zheng, Q.; Huo, Y.; Xie, F.; Zhao, J.; Lin, D. An efficient electrolyte additive of tetramethylammonium sulfate hydrate for Dendritic-Free zinc anode for aqueous Zinc-ion batteries. *J. Colloid Interface Sci.* **2022**, *627*, 367–374. https://doi.org/10.1016/j.jcis.2022.07.081.

[28] Xin, T.; Zhou, R.; Xu, Q.; Yuan, X.; Zheng, Z.; Li, Y.; Zhang, Q.; Liu, J. 15-Crown-5 ether as efficient electrolyte additive for performance enhancement of aqueous Zn-ion batteries. *Chem. Eng. J.* **2023**, *452*, 139572. https://doi.org/10.1016/j.cej.2022.139572.

[29] Liu, Z.; Wang, R.; Ma, Q.; Wan, J.; Zhang, S.; Zhang, L.; Li, H.; Luo, Q.; Wu, J.; Zhou, T.; et al. A Dual-Functional Organic Electrolyte Additive with Regulating Suitable Overpotential for Building Highly Reversible Aqueous Zinc Ion Batteries. *Adv. Funct. Mater.* **2023**, *34*, 2214538. https://doi.org/10.1002/adfm.202214538.
